# Advanced Therapeutic Options for Treatment of Metastatic Castration Resistant Prostatic Adenocarcinoma

**DOI:** 10.3389/fphar.2021.728054

**Published:** 2021-11-10

**Authors:** Faiza Naseer, Tahir Ahmad, Kousain Kousar, Sadia Anjum

**Affiliations:** ^1^ Industrial Biotechnology (IBT), Atta-ur-Rehman School of Applied Biosciences (ASAB), National University of Science and Technology, Islamabad, Pakistan; ^2^ Basic Medical Sciences, Shifa Tameer e Millat University (STMU), Islamabad, Pakistan; ^3^ Department of Biology, University of Hail, Ha’il, Saudi Arabia

**Keywords:** prostatic adenocarcinoma, metastatic castration resistant prostate cancer, immunotherapeutic agents, oncolytic virotherapy of cancer, crispr/Cas9 mediated gene therapy

## Abstract

The initial stage of prostatic adenocarcinoma (PaC) has been treated with surgery and radiation therapy, but the advanced stages need systemic novel treatment. Since 2010, several advanced therapeutic innovations have been introduced in various randomized clinical trials to improve survival and reduce morbidity and mortality. Several of these therapeutics have shown substantial survival assistance globally, even in the advanced stages of metastatic castration-resistant prostatic adenocarcinoma (mCRPC). This article describes advanced PaC therapy regimens including chemotherapeutic options, hormonal therapies (abiraterone, enzalutamide), immunotherapeutic agents, and bone-modifying agents. We discussed various pros and cons of gene therapy approaches including Crispr/Cas9 mediation, oncolytic viruses, suicidal genes, and micro-RNA based antitumor therapy. The mCRPC microenvironment is characterized by elevated prostate-specific antigen (PSA) levels, which ultimately trigger the androgen receptor (AR) and its dependent signaling pathways. The advanced therapeutics target these receptors and inhibit the steroidogenic enzymes that play an important role in increasing testosterone (T) and dihydrotestosterone (DHT) levels in the body. These advanced therapeutic novelties also target AR-independent oncogenic signaling pathways by focusing on DNA damage repair (DDR) pathways and their mechanisms. Some of these options appear to be very attractive strategies for acute and chronic stages of PaC and mCRPC treatment by overcoming the mechanisms of resistance.

## Introduction

PaC is a male-specific cancer of the prostate gland, which serves as the leading cause worldwide associated with interrupted urine flow, urge to urinate frequently at night, blood in the urine and seminal fluid, erectile dysfunction, and enlarged prostate in men. Approximately 70–80% of patients with PaC develop bone metastases and regional lymph node metastasis is observed in 5–12% of patients (Maolake et al., 2018). It arises as an Androgen-driven disease. Therefore, the mainstay of systemic therapy for patients with the advanced stage of the disease is androgen deprivation therapy (ADT). If the disease is significantly mitigated by ADT, then it is considered as Castration Sensitive Prostate Cancer and accounts for 3% of all PaC. However, despite significant responses, nearly all patients ultimately progress, and castration resistance ensues. Metastatic castration-resistant prostate cancer (CRPC) is defined by consecutive rises in serum prostate-specific antigen (PSA) levels or/and progression of metastatic spread in the setting of castrate levels of testosterone due to the stage of cancer when it no longer completely responds to treatments that lower testosterone (Vlachostgrios et al., 2017).

**FIGURE 1:**
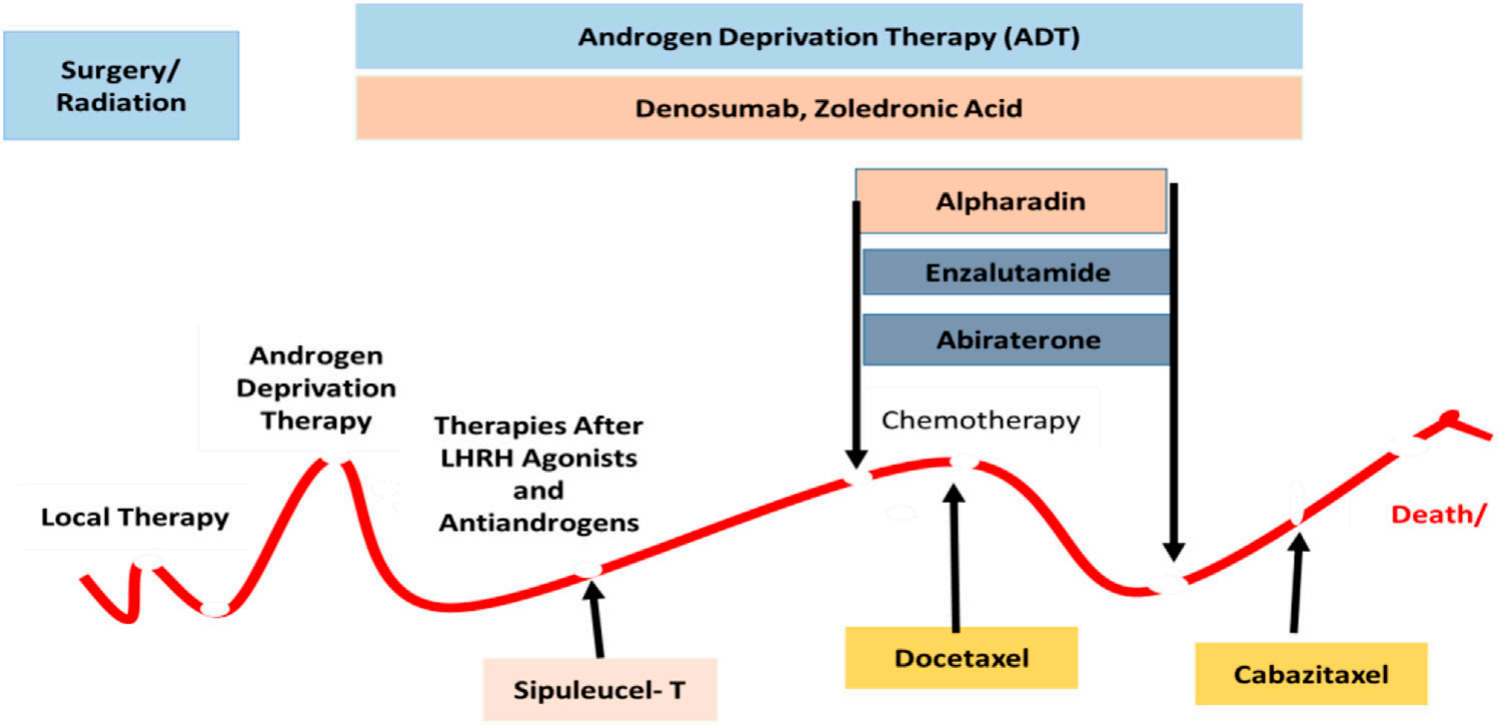
There are different types of treatment for patients with PaC depending upon the stage of disease displayed in the landscape. The standard treatments are included as active surveillance, Surgery, Radiation therapy and radiopharmaceutical therapy, Hormone therapy, Chemotherapy, and Immunotherapy. Combinations of different moieties have been used in the complex phase of the disease.

Its complexity and severity garnered interest amongst the scientific and medicinal communities and a wide variety of therapeutic approaches have been developed to tackle this disease, including complete castration. Patients older than 60 with a sluggish localized tumor may be placed on active surveillance, otherwise, doctors may go with a transurethral resection of the prostate (TURP), prostate artery embolization (PAE), and pelvic lymphadenectomy, with or without radiation therapy. The available pharmacological treatment options are clearly displayed in Figure 1.

## Androgen Deprivation Therapy

In the past, men diagnosed with mCRPC along with increased androgen levels have been treated with androgen deprivation therapeutics, but castration resistance has been observed in all patients which were subsided by advanced therapy that could include additional AR axis targeted moieties such as abiraterone and enzalutamide chemotherapy (Litwin and Tan, 2017).

### Androgen Pathway Inhibitor

Androgen antagonists that target the LHRH receptor represent the first two generations of androgen deprivation therapeutics. Next-generation moieties include abiraterone, enzalutamide, and apalutamide, which present a broader spectrum against mCRPC than previous options (Howard et al., 2019).

#### Abiraterone

**FIGURE 2:**
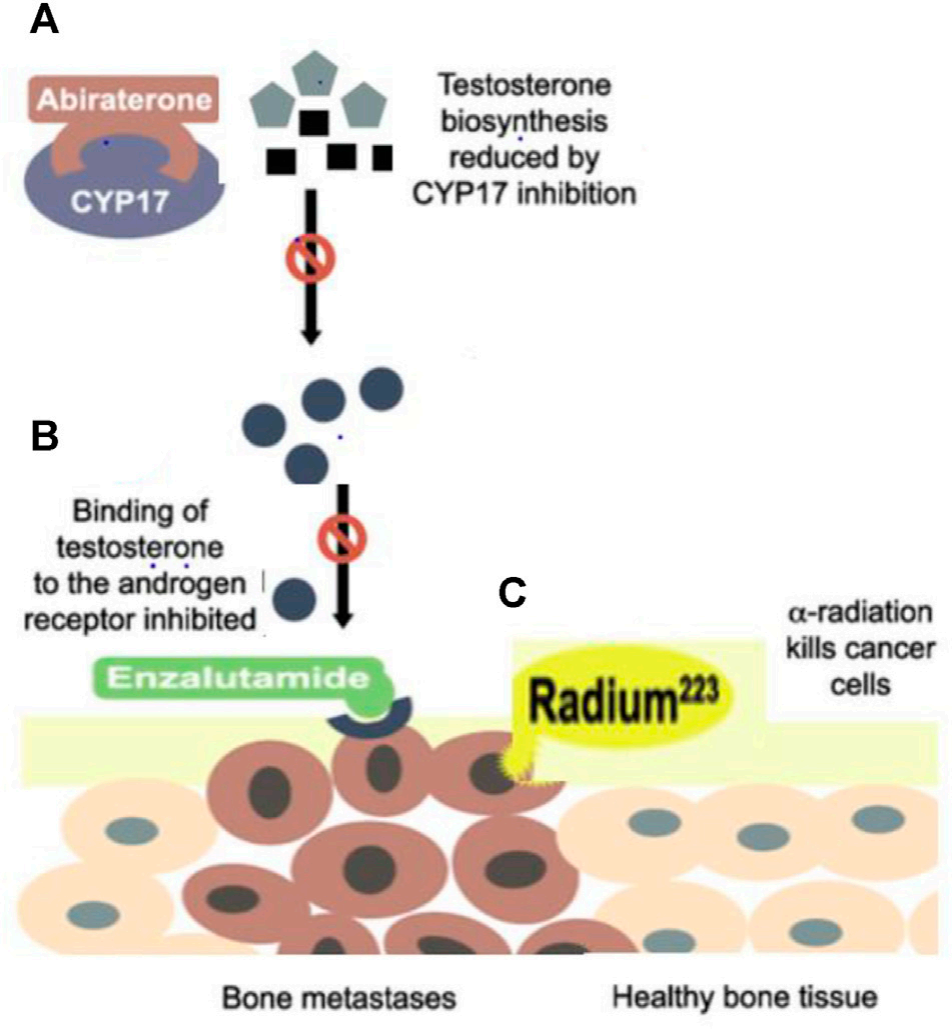
**(A)** Abiraterone inhibits the CYP17A which is required for 17a-hydroxylase and C17, 20-lyase activities cause a reduction in the biosynthesis of androgens in the adrenal glands and the testes and also blocks Testosterone production **(B)** Enzalutamide blocks androgen activity inside the cell, averting nuclear translocation, nucleic acid attachments, and transcription by blocking AR and also prevent transcription by interacting at the DNA binding site **(C)** Radium-223 penetrates in cancerous bones but causes less damage to healthy bones.

Abiraterone acetate (AA) is an orally administered irreversible, highly selective CYP17A inhibitor that targets its 17a-hydroxylase and C17, 20-lyase activities resulting in decreased biosynthesis of androgens in the adrenal glands and the testes. It also blocks T cells production (Nuhn et al., 2019), as shown in Figure 2. It has been administered with prednisolone and ADT agents, resulting in a reduction of whole-body sources of androgen production including the testes, adrenal glands, and PaC cells’ microenvironment. It has been observed in-patient with mCRPC whose disease progressed after the use of docetaxel, Abiraterone has increased the radiographic progression-free survival (rPFS) by 2 months and overall survival (OS) by 3.9 months approximately as compared to docetaxel-naïve patients, rPFS increased by 8.3 months and OS by 4.4 months. When abiraterone is added with ADT, testosterone is also reduced by distinct and complementary pathways and ultimately enhances the required outcomes with the suggestion that testosterone has the capacity to propagate the PaC cells, even at low levels below 20 ng/dl. Therefore, reduction of testosterone levels to zero can bring additional encouraging assistance to treatment for mCRPC patients (Gartrell and Saad, 2015).

The supposed AEs (adverse effects) with a low testosterone level are mineralocorticoid toxicity including high blood pressure, low potassium level, and edema with hepatic abnormalities, which sometimes may lead to fulminant hepatitis and acute liver cirrhosis have seen with the administration of abiraterone. Due to the associated administration of steroids, other AEs are CNS disturbances, headache, nausea, and vomiting. But both drugs in combination considerably reduce tumor burden and mortality, also provide additional clinical benefits in mCRPC patients (Fizazi et al., 2017).

ADT with gonadotropin-releasing hormone (GnRH) antagonist as degarelix is used in support of advanced PaC treatment. These chemical moieties decrease the levels of luteinizing hormone (LH) and follicle-stimulating hormones (FSH), thereby lowering testosterone to castrate levels, but along with this, adverse events including cardiovascular disorders, bone fractures, metabolic dysfunction, and impaired cognitive function have been observed (Shore et al., 2013).

#### Enzalutamide

Enzalutamide is an oral, second-generation, first-line nonsteroidal antiandrogen moiety specially formulated for a positive therapeutic effect in mCRPC men by binding to the AR at specific androgen binding region. So, the main target of enzalutamide is AR by blocking the effects of androgens inside the cell, averting translocation inside the nucleus, nucleic acid attachments, and transcription without activating AR, and also preventing transcription by interacting at the DNA binding site as shown in Figure 2 (Gravis, 2019). This prevention of AR-dependent transcription roots the decline in cell proliferation and boosts apoptosis. Enzalutamide antagonizes testosterone effects inside the cell regardless of where it is derived and is administered in addition to ADT (Crawford et al., 2019).

It had been observed in patients with mCRPC who had received enzalutamide as compared to a placebo before the administration of chemotherapeutic agents, median OS improved from 13.6 to 18.4 months. Regarding the safety of this chemical moiety, along with expected AEs, seizures and posterior reversible encephalopathy syndrome have been rarely observed because the chemical moiety has the ability to reach cerebrospinal fluid in CNS (Saad, 2013).

#### Apalutamide

Apalutamide is a second-generation androgen receptor inhibitor approved for the treatment of patients with non-mCRPC and metastatic castration-sensitive prostate cancer. It blocks the effect of androgens on the tumor. Specifically, it binds to the ligand-binding domain of the androgen receptor, blocks androgen-receptor nuclear translocation, inhibits DNA binding, and obstructs androgen receptor-mediated transcription. Phase I and II trial experience demonstrates the safety and tolerability of apalutamide, as well as its efficacy in effecting prostate-specific antigen response and radiographic-free survival in CRPC (Chong et al., 2018).

#### Darolutamide

**FIGURE 3:**
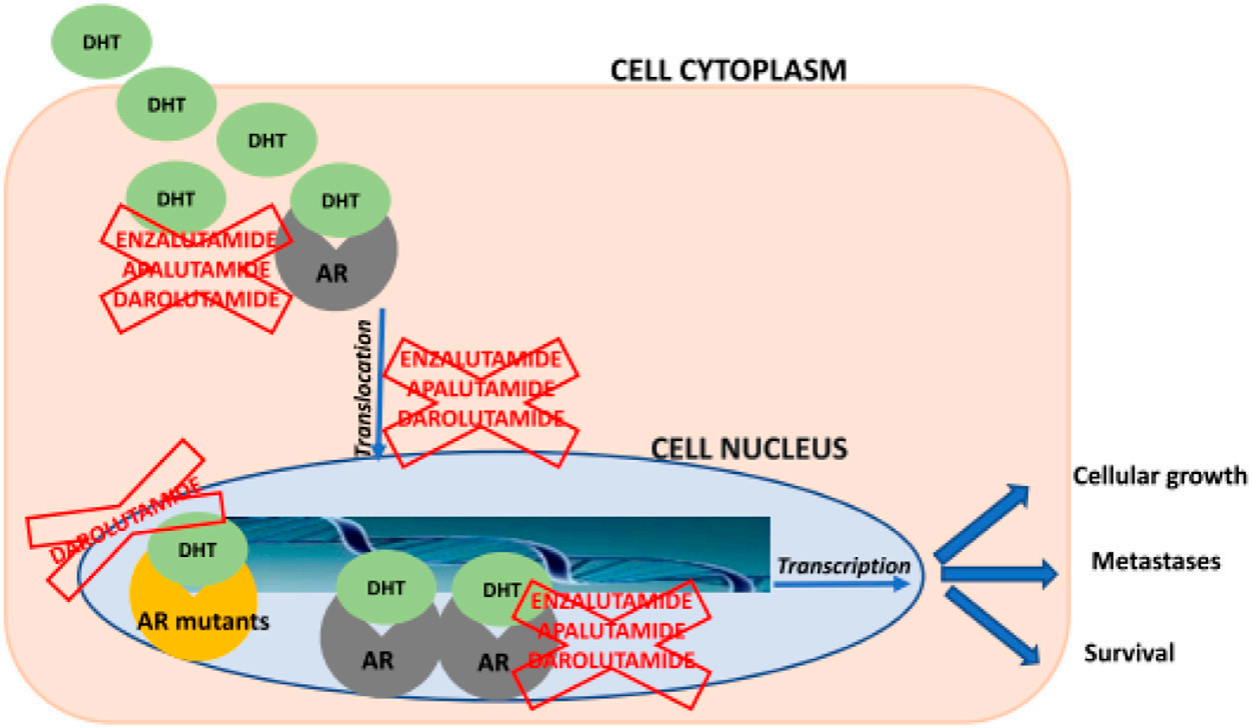
Schematic overview about mechanisms of action of enzalutamide, apalutamide, and darolutamide. AR = androgen receptor; DHT = dihydrotestosteron (Heidegger et al., 2020).

A new nonsteroidal investigational moiety related to AR antagonist is Darolutamide and formulated for oral administration, unlike all other advanced investigational moieties. The structure of the parent drug and its active metabolites are different from current second-generation antiandrogens including enzalutamide. This drug has shown high affinity to AR and damages following androgen-induced nuclear translocation of AR and transcription of the targets of AR genes upon strong binding at its specific binding region after conformational configuration as shown in Figure 3. Darolutamide has proved it has cancer cell killing potential by showing lethal reduction of cell proliferation in different cell lines obtained from mCRPC patients including AR mutants and prominent inhibition of tumor cell burden and number in xenograft mouse models as compared to second-generation antiandrogen agents (Shore, 2017). During clinical trials, no data has been reported about seizures or the risk of seizures even in those patients having a previous history of seizures. Furthermore, darolutamide has shown a greater safety and tolerability profile among mCRPC patients due to its least penetration in BBB upon administration *via* enteric or parenteral route. The most common treatment-related adverse effect reported was a lethargic body state.

Darolutamide has been reported as the most favorable chemical moiety activity against metastatic tumors in CYP17 inhibitor-naïve patients in combination with chemotherapy during open-label multicenter phase I/II trial in mCRPC patients. Results of two ongoing randomized, double-blind, placebo-controlled Phase III trials of darolutamide are pending, evaluating treatment in men with high-risk mCRPC and men with mHSPC (metastatic hormone-sensitive prostate cancer) (Ganapathy et al., 2020).

### Targeted Therapy

#### PARP Inhibitors (Olaparib)

An advanced therapeutic strategy for chemotherapeutic resistant levels of breast and ovarian cancers, especially when BRCA1/2 or other germline genetic material mutation defects are included in etiology, is Poly(ADP–ribose) polymerase (PARP) inhibition. These chemical moieties show synergism with androgen inhibitors and immunotherapeutic agents and have the ability to repair the damaged genetic material and prevent the diseased cells from repairing, so that those cells lead to mortality. It was observed in one cohort study that when olaparib was administered to mCRPC patients as compared to enzalutamide or abiraterone with a past history of using second-generation hormonal therapeutics and one taxane chemotherapeutic agent, radiographic progression-free survival (rPFS) was prominently enhanced in the olaparib with an rPFS of 7.4 compared to 3.6 months with other drugs (de Bono et al., 2020).

Large-scale multicenter efforts recently demonstrated germline genetic material mutation defects in up to 11.8% of men with advanced PaC. A comparable proportion of mCRPC anchorage somatic alterations were found in these genes as well, suggesting the potential benefit of PARP inhibition in PaC (Teo et al., 2019). These agents are well toleratable upon administration but cause mild hematological and gastrointestinal-related disorders.

#### Pembrolizumab

In recent times, the humanized, anti-PD-1 monoclonal antibody pembrolizumab has proved its pharmacological efficacy against tumors with lesser morbidity and pronounced safety in mCRPC patients. Those patients were previously treated with chemotherapeutic agents including docetaxel and one or more targeted endocrine therapies, with approximately one-fourth of the patients receiving both enzalutamide and abiraterone moieties (Antonarakis et al., 2020). In May 2017, the US Food and Drug Administration (FDA) established augmented endorsement to pembrolizumab for metastatic malignant tumors including microsatellite instable-high (MSI-H) or mismatch repair-deficient (dMMR) solid neoplasia that worsened after treatment with conventional chemotherapeutic agents, with higher morbidity and mortality rates and no satisfying current treatments strategies. This was the first chemical moiety approved for use based on a molecular biomarker rather than outdated diagnostic techniques based on the histopathological data of critical patients (Tucker et al., 2019).

### Cytotoxic Taxane Chemotherapy

#### Cabazitaxel

An advanced second-generation semisynthetic tubulin-binding taxane derivative is cabazitaxel. The administration of this chemical moiety causes high improvement in OS compared with mitoxantrone in combination with steroidal agents in mCRPC men whose disease has become worse with or after docetaxel administration in the TROPIC phase III, trial with a median survival of 15.1 months in the cabazitaxel patients and 12.7 months with the mitoxantrone use. Cabazitaxel sees its cytotoxic potential against mCRPC progres after treatment with docetaxel and abiraterone or enzalutamide or their related chemical moieties (Nuhn et al., 2019). The reported AEs were febrile neutropenia, diarrhea, blood-related infection, and more frequent blood in urine (Teo et al., 2019).

The administration of this tubulin-binding taxane can be more promising for patients with a high neutrophil count, advanced age, poor PS, previous episodes of febrile neutropenia, wide-ranging previous radiotherapy, deprived dietary habits, or other serious comorbidities. The FIRSTANA study recently showed significant data on doses of cabazitaxel that 20 mg/m^2^ was less harmful and as effective as 25 mg/m^2^ of cabazitaxel with lesser adverse effects, so 20 mg/m^2^ without GCSF support is referred to as an effective dose (Komura et al., 2018).

### Immunologic Moiety (Vaccine)

Moieties that could successfully hide from the immune system caused a complimentary cascade in PaC, helping in the early detection of the disease and boosting the lethal effect on tumor cells by directly killing them and activating the body’s own immunological mechanisms, known as immunotherapeutic approaches. Hiding from the immune system is supported by immune-suppressive cells i.e. regulatory T cells and myeloid-derived suppressor cells, soluble factors IL-6, IL-10, vascular endothelial growth factor, and transforming growth factor beta, and various active immune checkpoints. Immunologic moieties are formulated to boost or reactivate the defense mechanism against tumor cells (Nuhn et al., 2019).

#### Sipuleucel-T

The first cell-based vaccine is formed from the activation of a patient’s own antigen-presenting cells and their precursors from outside the body in an artificial culture media with cancer cell surface-specific tumor-associated antigens and then viable cells parenterally administered to the patients. On the basis of this phenomenon, sipuleucel-T was the first immunotherapeutic agent against prostate cancer which is now approved by the US Food and Drug Administration (Quinn et al., 2015).

Sipuleucel-T belongs to the autologous dendritic cell vaccine class. The leukapheresis is used to collect the mononuclear cells from peripheral blood, incubated with recombinant fusion protein with prostatic acid phosphatase and granulocyte-macrophage colony-stimulating factor for reactivation of immune cells and then viable cells administered to the patients with the schedule of three parenteral infusions after approximately 14 days intervals for 3 months (Gravis, 2019). This therapeutic cancer vaccine is the first and to date only immunotherapeutic that has proved to have positive effects on survival along with lesser morbidities among mCRPC men with asymptomatic or minimally symptomatic. Sipuleucel-T administration is 3 scheduled infusions to be administered every 14 days, which are highly individualized and generated for each patient accordingly.

In 2019, the phase III IMPACT trial showed a 4.0 MO improvement in median survival with 25.8 MO in the sipuleucel-T group as compared to 21.7 in the placebo group, in total 512 patients randomly assigned in a 2:1 ratio (sipuleucel-T *vs.* placebo) (Nuhn et al., 2019). The observed adverse effects reported are chills, pyrexia, and headache which are comparable with other chemotherapeutic/hormonal agents with lesser morbidity (Komura et al., 2018).

### Bone Targeted Therapy

The mCRPC most often spreads towards bone and symptomatic treatment is commonly done by bisphosphonates, external beam radiation, and denosumab, a monoclonal antibody therapy used for the treatment of osteoporosis and contraindicated in hypocalcemia.

#### Radium-223

Radioisotopes such as samarium-153 and strontium-89 have been previously reported as therapeutic options, either alone or in combination with chemotherapeutic moieties for the treatment of different stages of mCRPC men. No survival benefit has been shown with the usage of radioisotopes. They are limited to offering symptomatic treatment, especially in men with metastatic disease. However, these isotopes emit beta rays upon administration, which is very toxic for bone marrow-related diseases.

Thus, a new approach has been introduced, an alpha-emitter radioactive parenteral formulation which can penetrate directly deep into osteocytes with less damage and diffusion to healthy cells, including bone marrow, erythrocytes, and leukocytes, and is used to treat mCRPC with fewer hematological disorders as shown in Figure 1. Radium 223 has no effect on lowering PSA levels so, its main mechanism of action is similar to Ca by interacting with the microenvironment of sclerotic metastases with a significantly slighter series than beta radiations (Komura et al., 2018). When the damage has already spread to lymph nodes, lungs, or hepatic cells, this therapy is not recommended. The chance of fracture increases if Radium-223 is used with abiraterone and prednisolone (Gravis, 2019).

### Gene Therapy Approaches

#### CRISPR/Cas9 Mediated Treatment

**FIGURE 4:**
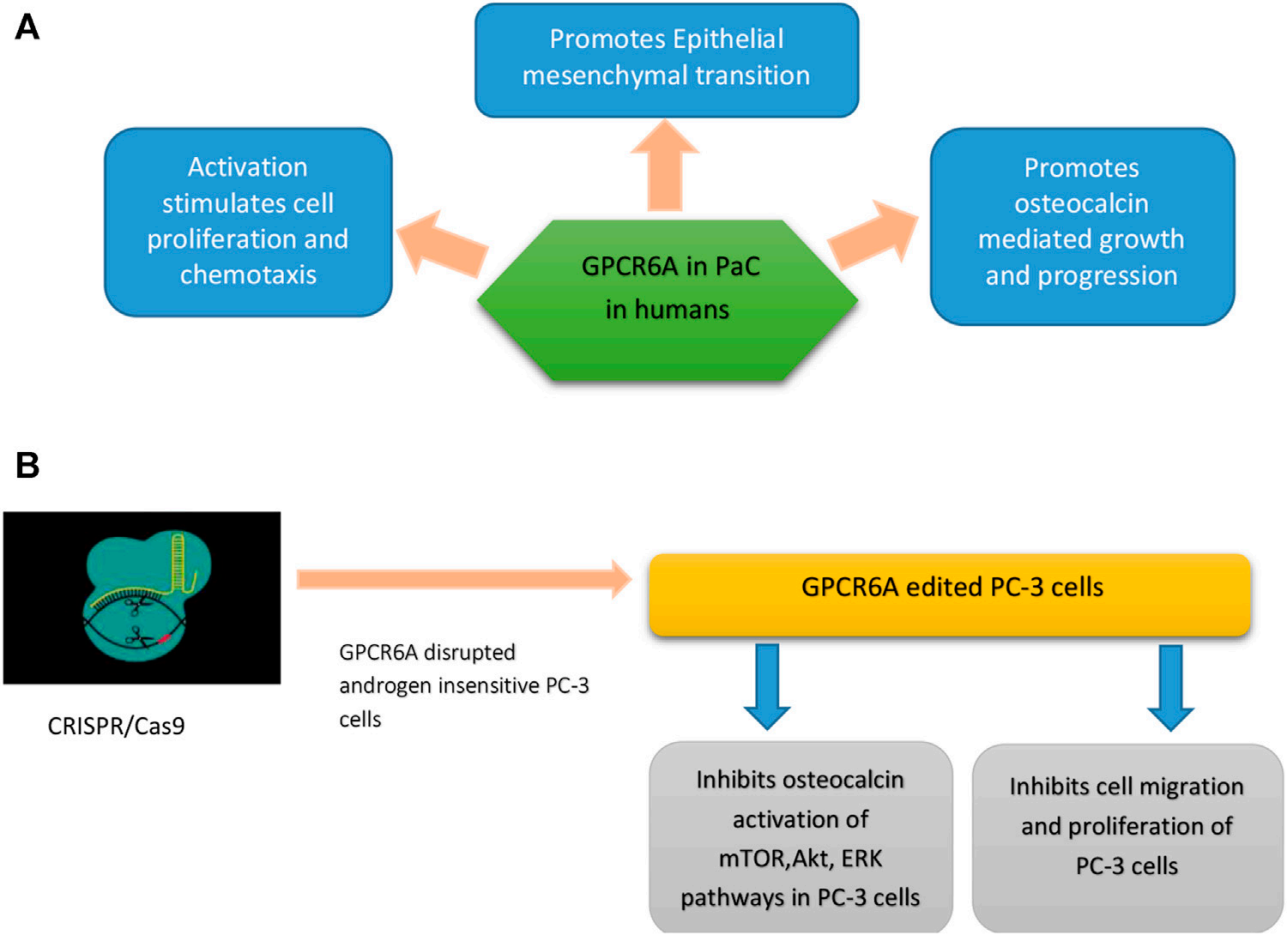
**(A)** Role of GPCR6A in PaC in humans (a result of RKLP to KGKY evolutionary polymorphism) **(B)** GPCR6-A disrupted androgen insensitive PC-3 cells show tumor regression and inhibition of osteocalcin mediated mTOR, AKT, and ERK signaling pathways.

CRISPR/Cas9, a biotechnological methodology has been implicated in gene knock-in, knock-out, gene silencing, and suicide gene delivery. Its mediated gene disruption demonstrated significant involvement of GPRC6A in the development of cancer and progression to mCRPC. GPRC6A is an extensively pronounced G protein-coupled receptor and is the principal regulator of sexual reproduction and energy metabolism (Ye et al., 2017). There are many ligands for GPCR6A but testosterone and osteocalcin are of major concern when it comes to the development and pathogenesis of mCRPC. PaC produces factors that disturb the normal functional balance between osteoblast and osteoclast in bone, thus overall affecting the bone microenvironment. Bone marrow produces osteocalcin that has been implicated in mCRPC development (Wong et al., 2019). The activation of GPCR6A through autocrine or paracrine discharge of osteocalcin may increase the development of PaC cells (Hayashi et al., 2016) as shown in Figure 4.

GPCR6 is also involved in the release of hormones like adiponectin from adipocytes, IL-6 from myocytes, insulin from B cells, and testosterone from Leydig cells (Clemmensen et al., 2014). In humans, a KGKY polymorphism in the third intracellular loop (ICL3) that replaces ancestral RKLP in GPCR6A is proposed to increase the onset and pathogenicity of PaC (Jørgensen et al., 2017). For this purpose, research was conducted which involved a comparison of humanized mouse GPRC6A with mouse wild-type GPRC6A, where both receptors were expressed on the cell surface. It found that humanized GPRC6A preferentially activated mToR-signaling pathways largely leading to aggressive prostate tumor development, as compared to mouse (RKLP) GPCR6A that also activated m-ToR signaling in HEK-293 cell lines.

Through CRISPR/Cas9, endogenous GPCR6A was disrupted in androgen insensitive human PC-3 cells. These amended PC-3 cells showed reduced reaction to testosterone and osteocalcin mediated activation of Akt, ERK, and M-ToR phyophorylation (advanced PaC progression pathways) as compared to controls, which had normal GPCR6A expression. CRISPR/Cas9 mediated GPRC6A deficient PC-3 xenograft cells showed reduced tumor growth and osteocalcin-associated progression of prostate cancer as compared to GPRC6A expressing PC-3 cells (Ye et al., 2017).

#### Oncolytic Virus Immunotherapy

**FIGURE 5:**
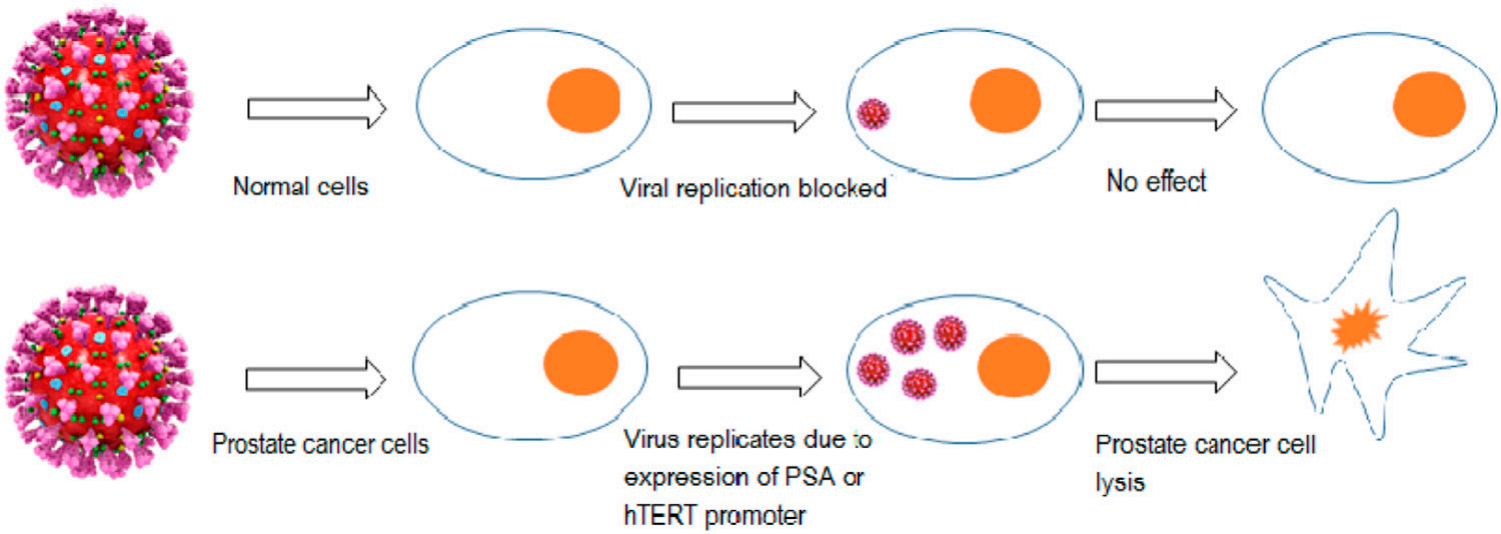
Targeted replication of the oncolytic virus in only PaC cells under tissue-specific promoter (PSA promoter) or due to the presence of an overly expressed enzyme (human telomerase reverse transcriptase).

One approach to treat mCRPC is to use replication-competent viruses that can act as a vehicle of gene delivery or can specifically target cells with overexpressed receptors associated with oncogenicity and suppress metastatic castration-resistant prostate cancer. The specificity of this approach can be enhanced by either i) Inducing a mutation to allow the virus to replicate in only those cells harboring a specific mutation i.e. p53 mutation ii) By developing viruses that would replicate only under a definite tissue-specific promoters, such as the promoter for PSA or prostate-specific membrane antigen (PSMA) as they are overly expressed in PaC. The promoter for human telomerase reverse transcriptase (hTERT) was used for the purpose of selectively inducing transgene expression of modified oncolytic virus in cancer cells that express hTERT enzyme (Fukuhara et al., 2009) as shown in Figure 5. In a phase I clinical trial an adenovirus AdhTERTp-E1A was created that would replicate conditionally in cells where the hTERT promoter regulates the E1A viral gene. This restricted the replication of AdhTERTp-E1A to only those tumor cells that are telomerase positive. Intra-tumoral injections AdhTERTp-E1A given to athymic mice having prostate or liver cancer xenograft showed noticeable inhibition in tumor growth, it even exhibited complete tumor suppression in some cases. These effects were observed without any toxicity effects (Irving et al., 2004).

#### Suicide Gene Therapy

Suicide gene therapy is also called Gene-directed enzyme prodrug therapy (GDEPT). For PaC treatment, the extensively used gene-directed enzyme systems based on prodrug therapy include i) Cytosine deaminase and 5 flourocytosine ii) HSV thymidine kinase and ganciclovir or acyclovir (Zhang et al., 2015). An oncolytic adenovirus (Ad5-CD/TKrep) was developed that had a CD/HSV-tk fusion gene. The replication-competent Ad5-CD/TKrep leads to enhanced transgene expression in cancer cells as compared to non-replication competent adenovirus (up to 2000-fold per cell). The intra-tumoral administration of Ad5-CD/TKrep was evaluated in Phase I trial, in combination with prodrugs ganciclovir and 5-fluorocytosine (5-FC). Ad5-Cd/TKrep lead to a significant reduction in tumor with a good safety profile. More than a 25% decrease in PSA was observed in 44% of patients, whereas more than 50% decrease in PSA was seen in 19% of patients. The killing of tumor cells was observed by biopsies at the tumor administration sites. Two patients were found found to be completely cancer-free at a one-year follow-up (Barton et al., 2006).

**FIGURE 6:**
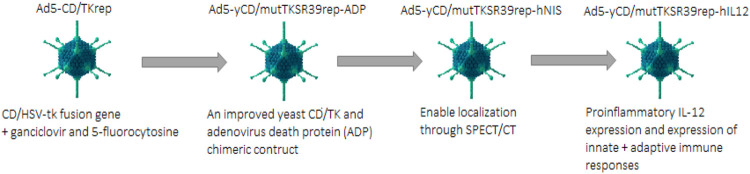
Developmental regimen of adenovirus for the treatment of PaC.

In phase II clinical trial Ad5-yCD/mutTKSR39rep-ADP was constructed which contained an improved yeast CD/TK and adenovirus death protein (ADP) chimeric construct. This Ad5-yCD/mutTKSR39rep-ADP showed greater cancer-killing capacity and even promising tumor regression was observed when this therapy is used in synergy with radiation therapy (Freytag et al., 2014). Another version, Ad5-yCD/mutTKSR39rep-hNIS, was created from the mutant Ad5-yCD/mutTKSR39rep-ADP. This version contained a reporter gene human sodium iodide symporter in addition to the suicide gene, which enabled localization through noninvasive single-photon emission computed tomography (SPECT/CT) (Ali & Halldén, 2018) as shown in Figure 6.

The recent version of this mutant Ad5-yCD/mutTKSR39rep-hIL12 expresses human IL-12. Expression of proinflammatory IL-12 has been reported as having antitumor activity and is usually expressed by antigen-presenting cells to induce innate and adaptive immunity; this construct Ad5-yCD/mutTKSR39rep-hIL12 induced a proinflammatory response in addition to its normal oncolytic activity. This construct expressing Il-12 helps oppress the tumor supporting microenvironment and inhibits the process of angiogenesis. Administration of Ad5-yCD/mutTKSR39rep-hIL12 through local injection may help bypass the systemic toxicity effects of IL-12 while preserving its pharmacological efficacy at local administration (Barton et al., 2020).

#### Micro-RNA Based Antitumor Therapy

Micro RNAs can act as both tumor suppressor and oncogenes. Dysregulation of miRNAs are seen to be associated with the sustenance of cancer as dysregulation tends to activate metastasis, invasion, resist cell death and induce angiogenesis. Anti-tumor microRNAs are attractive prognostic markers and therapeutic targets for cancer.

#### MicroRNA-299-3p

AR signaling shows a significant function in the worsening of mCRPC. MicroRNAs play a pivotal role in the mediation of signaling pathways involved in tumor advancement or suppression. Recent studies have shown that miR-299-3p has the promising capacity to specifically target AR and regress tumor growth in mCRPC. Additionally, loss of miRNA-299-3 was observed in PaC cells as compared to normal prostate cells. An increase in expression of apoptotic markers, reduced proliferation, migration, and cell cycle arrest was observed when 22Rv-1, PC-3, and C4-2B cell lines replenished with miR-299-3. In addition, overexpression of miR-299-3p seen to increase expression of E-cadherin (Cell-cell adhesion receptors), expression of slug, and inhibition of epithelial-mesenchymal transition. Furthermore, overexpression of miR-299-3p in xenograft models lead to reduced tumor growth and increased sensitivity towards antitumor drugs (Ganapathy et al., 2020).

#### MicroRNA

It is short (20-24 nt), non-coding RNAs that are involved in the post-transcriptional regulation of gene expression in multicellular organisms by affecting both the stability and translation of mRNAs. The miRNA-205 is a promising biomarker for predicting the recurrence and progression of patients with adenocarcinomas or breast cancer. The development of an efficient miRNA delivery system is a highly challenging task due to the rapid degradation of miRNAs in serum conditions and low cellular internalization (Gardlik et al., 2005). Viral vector-based delivery of miRNA(s) has been conventionally used due to efficient transduction, however, its clinical translation is often limited by immunological and toxicological side effects. Moreover, limitation in transgenic capacity size and their expensive nature makes them a difficult choice. Previous studies have shown that miR-205 negatively regulates prostate cancer cell proliferation, metastasis, and drug resistance but a miR-205 nanoplatform induces significant apoptosis and enhances chemotherapeutic effects in prostate cancer cells (Nagesh et al., 2018).
